# Comprehensive analysis of *PTPN* family expression and prognosis in acute myeloid leukemia

**DOI:** 10.3389/fgene.2022.1087938

**Published:** 2023-01-09

**Authors:** Yong Liu, Jing Zhang, Zefan Du, Junbin Huang, Yucai Cheng, Wenfang Yi, Tianwen Li, Jing Yang, Chun Chen

**Affiliations:** ^1^ Division of Hematology/Oncology, Department of Pediatrics, The Seventh Affiliated Hospital of Sun Yat-Sen University, Shenzhen, China; ^2^ Department of Breast and Thyroid Surgery, Guangzhou Women and Children’s Medical Center, Guangzhou, China

**Keywords:** PTPNs, AML, bioinformatics, prognosis, biomarker, expression level

## Abstract

**Background:** Tyrosyl phosphorylation is carried out by a group of enzymes known as non-receptor protein tyrosine phosphatases (PTPNs). In the current investigation, it is hoped to shed light on the relationships between the expression patterns of *PTPN* family members and the prognosis of acute myeloid leukemia (AML).

**Methods:** *PTPN* expression was examined using GEPIA and GEO databases. To investigate the connection between *PTPN* expression and survival in AML patients, we downloaded data from the Broad TCGA Firehose and Clinical Proteomic Tumor Analysis (CPTAC) of the Cancer Genome Atlas (TCGA). We used quantitative real-time PCR (qRT-PCR) to confirm that essential genes were performed in clinical samples and cell lines. We then used western blot to verify that the genes expressed in the above databases were positive in normal tissues, AML patient samples, and AML cell lines. Next, we investigated associations between genome-wide expression profiles and *PTPN6* expression using the GEO datasets. We investigated the interactive exploration of multidimensional cancer genomics using the cBioPortal datasets. Using the DAVID database, a study of gene ontology enrichment was performed. The protein-protein interaction (PPI) network was created using the STRING portal, and the gene-gene interaction network was performed using GeneMANIA.

**Results:** Data from GEO and GEPIA revealed that most *PTPN* family members were linked to AML. Patients with leukemia have elevated levels of several *PTPN* members. All of the AML patients’ poor overall survival (OS, *p* < .05) was significantly linked with higher expression of *PTPN1*, *PTPN6*, and *PTPN7*. Additionally, clinical samples showed that the expression of *PTPN 6*, *PTPN 7*, *PTPN 13*, and *PTPN 14* was higher than normal in AML patients (*p* = .0116, *p* = .0034, *p* = .0092, and *p* = .0057, respectively) and AML cell lines (*p* = .0004, *p* = .0035, *p* = .0357, and *p* = .0177, respectively). Western blotting results showed that the expression of *PTPN6* in AML samples and AML cell lines was significantly higher than that in normal control samples.

**Conclusion:** Differentially expressed PTPN family members were found in AML. The prognosis of patients and *PTPN* gene expression were shown to be correlated. *PTPN6* is one of these members and may be used as an AML diagnostic and prognostic marker.

## Introduction

The most prevalent kind of acute leukemia in adults and the cause of the most significant number of leukemia-related fatalities each year in the United States is acute myeloid leukemia (AML), a heterogeneous hematologic malignancy characterized by the clonal growth of myeloid blasts in peripheral blood, bone marrow, and other organs (Tallman et al., 2019). In 2022, it is predicted that 20,050 people will be diagnosed with AML, and 11,540 people will pass away from the condition (Siegel et al., 2022). Despite improvements in AML treatment, such as the introduction of chemotherapy and other successful targeted medicines over the previous few decades, the 5-years relative survival rate increased from 6.2% in the mid- 1970s to 30% for those diagnosed from 2009 to 2015 (Lai et al., 2019). However, there are significant restrictions in the prognosis predicted by the existing biomarkers because of the clinical and molecular heterogeneity of AML (Patel et al., 2012). Therefore, it is vital to find reliable biomarkers that will allow for an earlier diagnosis and better, more specialized therapy of AML.

Complex phosphorylation and dephosphorylation networks are created when kinases and phosphatases, which carry out phosphorylation and dephosphorylation, are linked by their shared substrates or direct interactions. These networks are essential for controlling cellular functions (Li et al., 2013). Protein tyrosine phosphatases (PTPs) are a group of enzymes that catalyze the dephosphorylation of tyrosine residues (Chen et al., 2020). One hundred three genes encode PTPs, which are organized into four primary superfamily classes. The Human Genome Organization’s Nomenclature Committee has given each PTP member an official gene name (Ogino et al., 2007). There are 17 non-receptor PTPs in Class I of the most prominent family, known as PTPN, with a number, according to the literature (Alonso et al., 2004). More and more evidence points to the possibility that protein tyrosine kinases (PTKs) and protein tyrosine phosphatases (PTPNs) collaborate to control a wide range of cellular processes, including immune response, migration, metabolism, and proliferation and differentiation (Tonks, 2013; Yu and Zhang, 2018). The *PTPN* family numbers significantly influence various disorders, according to numerous research that has already been published. For instance, *PTPN22* restricts T-cell receptor-induced proliferation. It hinders naive T-cell activation and effector cell responses in response to low-affinity antigens (Salmond et al., 2015), and *PTPN12* expression is elevated in both stomach adenocarcinoma and cancer (Chen et al., 2020). Additionally, one study indicates that the deletion of *PTPN2* may enhance the therapeutic effectiveness of CAR-T cells in the treatment of breast cancer (Wiede et al., 2020). The cell cycle, apoptosis, and metastasis are all heavily regulated by *PTPN2*, which is a significant predictor of the prognosis of pancreatic cancer (Kuang et al., 2022). The *PTPN* genes are generally a promising prognostic and therapeutic target for cancer therapy due to this evidence. However, the distinct roles of *PTPN* family genes in AML have yet to be understood entirely.

Therefore, discovering oncogenes or tumor suppressors mediated by *PTPN* as potential pathways for predicting biomarkers may offer novel therapeutic approaches for treating AML. The difficulty comes from the fact that most *PTPN* genes’ variances in transcriptional levels, prognostic values, molecular roles, and biological processes have not yet been thoroughly understood in the context of AML disease. In order to thoroughly investigate the association between *PTPN* subtypes and the pathogenesis and progression of AML, we combed through some widely used databases as part of this work to further our understanding of AML.

## Materials and methods

### Ethics statement

The Seventh Affiliated Hospital of Sun Yat-Sen University’s Academic Committee approved this study, which was carried out following the guidelines outlined in the Declaration of Helsinki. Each patient signed informed consent. Since all the datasets were taken from published works, it was verified that written informed consent had been obtained for every one of them.

### Download and expression analysis of microarray data

The GEO database (http://www.ncbi.nlm.nih.gov/geo) is a public functional genomics data repository that downloads the GSE149237 microarray dataset (Jäger et al., 2021). This dataset was obtained by comparing five healthy HSPCs sequenced and compared to eight AML patient samples, and the study was conducted with GPL20301 Illumina HiSeq 4000 sequencing platform. Then, we performed a classification analysis on the mRNA expression values of the target genes. The filter conditions are *p*-value <0.05 and the absolute value of the difference (| log2 (Fold Change) |) > 1.

Download the data set GSE37642 and its corresponding platform file GPL96 (Affymetrix Human Genome U133A Array). The GSE37642 data set based on the GPL96 platform contains a total of 422 tissue samples (bone marrow mononuclear cells) of AML patients, and clinical information such as survival time, survival status, and whether *PTPN6* mutations occur in the samples are extracted.

### GEPIA dataset

GEPIA (Gene Expression Profiling Interactive Analysis) is a newly created interactive web server for evaluating the RNA sequencing expression data of 9,736 tumors and 8,587 normal samples from projects like the Genotype-Tissue Expression (GTEx) and the Cancer Genome Atlas (TCGA), using a regular processing pipeline. (http://gepia.cancer-pku.cn/). Customizable features offered by GEPIA include dimensionality reduction analysis, similar gene discovery, patient survival analysis, profiling based on cancer kinds or pathological stages, tumor or normal differential expression analysis, patient survival analysis, and similar gene detection (Tang et al., 2017).

### LinkedOmics dataset

In the software ecosystem, LinkedOmics (http://www.linkedomics.orglogin.php) is a brand-new and unique tool for sharing data from extensive cancer omics initiatives. To reduce duplication of effort, which is concentrated on the detection and interpretation of attribute connections, preprocessed and normalized data from the Clinical Proteomic Tumor Analysis (CPTAC) data portal and the Broad TCGA Firehose are used, completing the work of the already-existing cancer data portals (Vasaikar et al., 2018).

### Cell lines and cell culture

Four AML cells, HL-60, KG-1, THP-1, and MOLM-13, were purchased from American Type Culture Collection (Rockville, MD). HL-60 and KG-1 were cultured in IMDM medium (Invitrogen, Shanghai, China) supplemented with 20% fetal bovine serum (Biological Industries, Kibbutz Beit Haemek, Israel) and 100 units/ml penicillin and streptomycin. THP-1 and MOLM-13 were cultured in RPMI medium (Invitrogen, Shanghai, China) supplemented with 10% fetal bovine serum (Biological Industries, Kibbutz Beit Haemek, Israel) and 100 units/ml penicillin and streptomycin. Cells were incubated at 37°C in a humidified atmosphere of 95% air and 5% CO_2_, as described previously (Jin et al., 2015). The cells were confirmed to be mycoplasma-free routinely.

### RNA extraction, reverse transcription and quantitative real-time polymerase chain reaction (qRT-PCR)

To verify the expression of crucial genes in clinical samples and cell lines, we further verified the expression level of essential genes in blood monocytes of four newly diagnosed patients with AML (confirmed by WHO-AML criteria, excluding AML-M3 cases. Not receive treatment was received) and four AML cell lines (HL-60, KG-1, THP-1, and MOLM-13) using qPCR. Peripheral blood monocytes from four anonymous healthy volunteers were used as control samples.

According to g Trizol reagent and the manufacturer’s instructions, total RNA was extracted from cultivated cells (Takara Bio, Kusatsu, Japan). The expression of the indicated genes was examined using SYBR Premix Ex Taq TM II and PCR detection equipment from Bio-Rad in Hercules, California, United States. A quick all-in-one RT-Kit was used to create the cDNA (ES Science Biotech). The internal control gene GAPDH’s transcript levels were used to standardize transcription levels. The supplementary table displays the order of the primers. Triplicate analyses of each RNA sample were carried out.

### Reagents and antibodies

PTPN6 (Rabbit, 3759) and PTPN14 (Rabbit, 13,808) antibody was from Cell Signaling Technology (Beverly, MA). Antibodies against β-actin were from Sigma-Aldrich (Mouse, A5441, Shanghai, China). Antibodies against PTPN1(Goat, AF3954) was from Novus Biologicals (Littleton, CO). Antibodies against PTPN7(Goat, AF3954) was from Novus Biologicals (Littleton, CO). Antibodies against PTPN13 (Rabbit, PA5-72907) were from Thermo-Fisher Scientific (Shanghai, China). The fluorescent-conjugated secondary antibodies anti-mouse and anti-rabbit IgG were from LI-COR Biotechnology (Nebraska, United States).

For western blotting assays, whole cell lysates were prepared in RIPA buffer (1 × PBS, 1% NP-40, .5% sodium deoxycholate, .1% SDS) supplemented with 10 mmol/L β-glycerophosphate, 1 mmol/L sodium orthovanadate, 10 mmol/L NaF, 1 mmol/L phenylmethylsulfonyl fluoride, and 1 × Roche complete Mini protease inhibitor cocktail (Roche, Indianapolis, IN) (Jin et al., 2017). The cytosolic fractionations for cytochrome c detection were prepared with digitonin extraction buffer (10 mmol/L PIPES pH 6.8, .015% digitonin, 300 mmol/L sucrose, 100 mmol/L NaCl, 3 mmol/L MgCl2, 5 mmol/L EDTA, and 1 mmol/L phenylmethylsulfonyl fluoride) as described previously (Jin et al., 2017). Protein samples were separated by SDS-PAGE and transferred to nitrocellulose membranes, which were then incubated with the primary antibodies. After incubation with appropriate secondary antibodies, the membranes were scanned by the Odyssey infrared imaging system (LI-COR, Lincoln, Nebraska).

### TCGA data and the cBioPortal

The cBioPortal (http://www.cbioportal.org/) for cancer genomics is an open-access and open-source platform developed for the interactive study of multidimensional cancer genomics datasets (Cerami et al., 2012). It supports and maintains data about non-synonymous mutations, DNA copy-numbers, mRNA and microRNA expression, protein-level and phosphoprotein levels, DNA methylation, and de-identified clinical data. We may compute mRNA expression z-scores (RNA Seq V2 RSEM), PTPN family gene correlations, and the frequency of gene modifications using the web tool cBioPortal.

### GO and PPI analysis for the function and interaction of PTPN family

Enrichment analysis of gene ontology (GO) of *PTPN* genes was explored using the Database for Annotation, Visualization and Integrated Discovery (DAVID; v.6.8; https://david.ncifcrf.gov/home.jsp; accessed on 20 November 2019) (Chandrasekharan et al., 2013). The gene-gene interaction network was structured using the Gene Multiple Association Network Integration Algorithm (GeneMANIA; https://www.genemania.org/; accessed on 21 November 2019) (Warde-Farley et al., 2010) and the search tool for the Retrieval of Interacting Genes Database (STRING v.10.0; https://string-db.org/; accessed on 23 November 2019) was used to create a protein-protein interaction (PPI) network (Szklarczyk et al., 2017).

### Statistical analysis

Utilizing the software packages R Studio (R version 4.0.2) and GraphPad Prism 8.3, statistical analysis and visualization were carried out (GraphPad Software, Inc., La Jolla, CA, United States). Single e-variable Cox proportional regression models and two-way ANOVA analysis were employed to examine the overall survival and mRNA expression datasets. An illustration of the prognosis was a Kaplan-Meier survival curve. We compared variations in central gene expression levels using two-way ANOVA analysis. Statistical significance was set at as < .05.

## Results

### Transcriptional level of *PTPNs* in patients with AML in the GEO database

**FIGURE 1:**
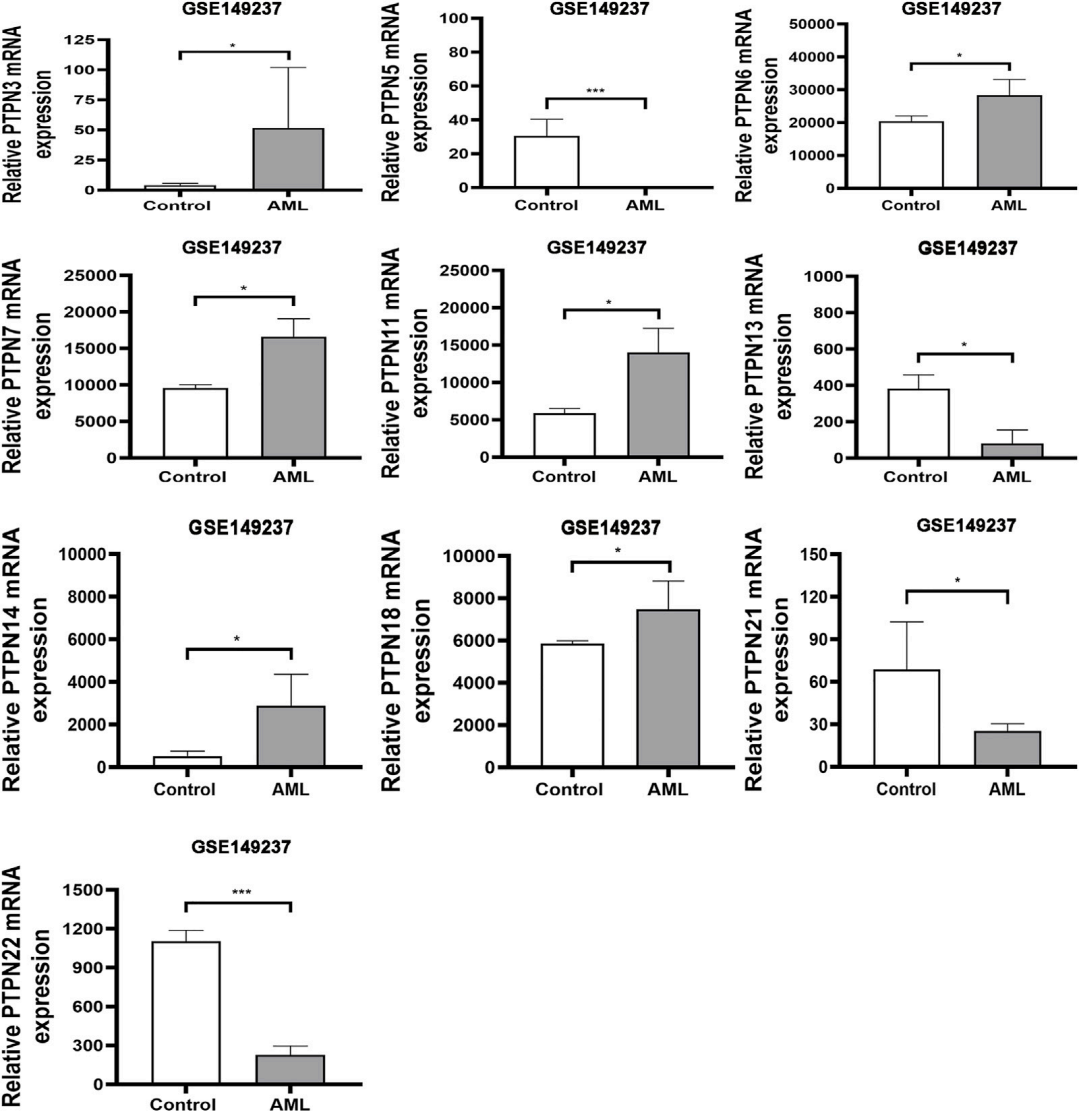
In the GSE149237 microarray dataset, there were differential transcript levels of the PTPN gene in AML and healthy control samples. The filter conditions are *p*-value <0.05 and the absolute value of the difference (| log2 (Fold Change) |) > 1.

The human genome contains *PTPN* genes, which have been found. We compared the transcriptional expression of the PTPN genes in tumorigenic and healthy control samples using the GSE149237 microarray dataset. GEO analysis revealed that the mRNA expression level of *PTPN3*, *PTPN6*, *PTPN7*, *PTPN11*, *PTPN14*, and *PTPN18*, was upregulated in AML patients compared with normal controls (*p* < .05, Figure 1). However, the expression levels of *PTPN5*, *PTPN13*, *PTPN2*1, and *PTPN22* were lower in AML patients (*p* < .05, Figure 1). While others, such as *PTPN1*, *PTPN2*, *PTPN9*, *PTPN12*, *PTPN20*, and *PTPN23*, showed no difference between AML and normal samples (*p* > .05, Supplementary Figure S1).

### The mRNA levels of *PTPNs* in AML samples and normal samples in the GEPIA

#### Database

**FIGURE 2:**
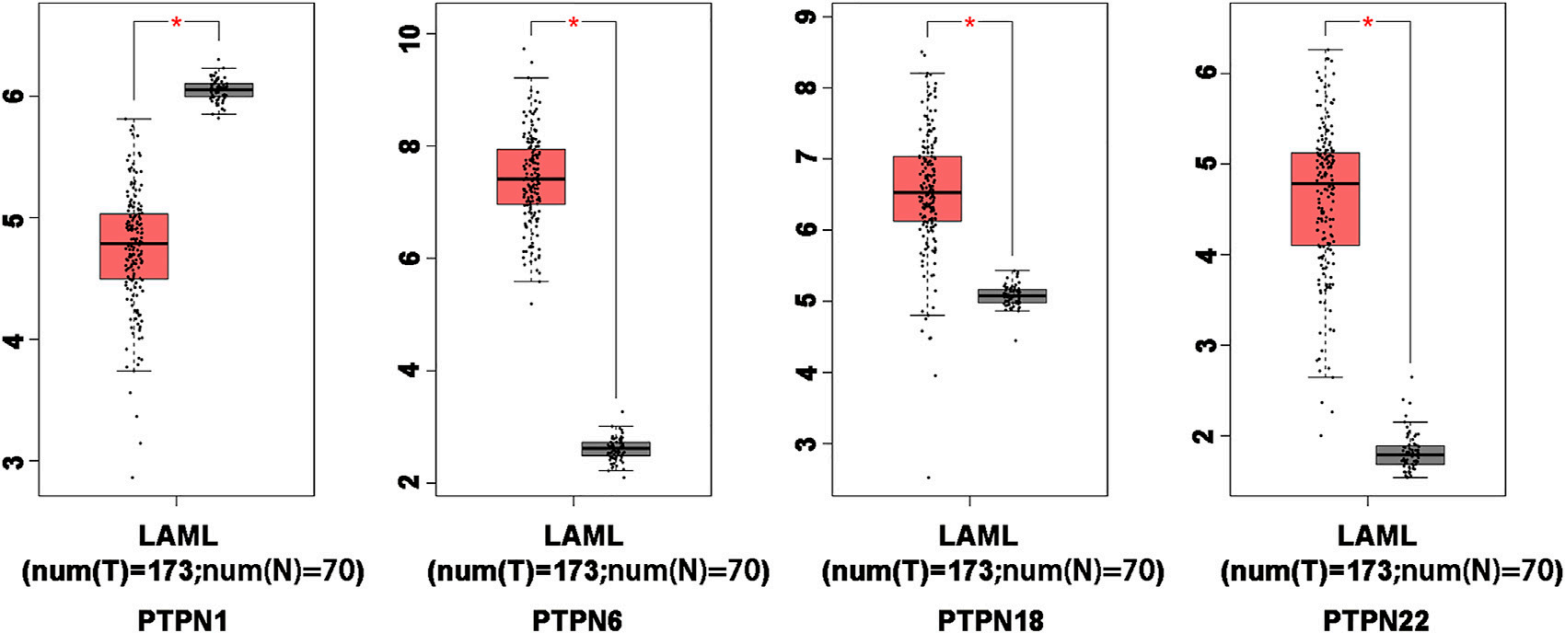
Differentially expressed PTPN genes in AML and healthy control samples in the GEPIA database. The filter conditions are *p*-value <0.05 and the absolute value of the difference (| log2 (Fold Change) |) > 1.

In order to compare the mRNA expression of *PTPN* factors in leukemia and normal samples, we used the GEPIA (Gene Expression Profiling Interactive Analysis) dataset (http://gepia.cancer-pku.cn/). The findings showed that *PTPN6, PTPN18*, and *PTPN22* had higher expression levels in leukemia than in normal blood samples, while *PTPN1* had a lower expression level in the former than the latter (Figure 2). However, there was no discernible difference in the expression levels of *PTPN2, PTPN3, PTPN4, PTPN5, PTPN7, PTPN9, PTPN 11, PTPN12, PTPN13, PTPN14, PTPN20, PTPN21*, and *PTPN23* (Supplementary Figure S2).

### Relationship between the mRNA level of *PTPNs* and the prognosis of patients with leukemia

**FIGURE 3:**
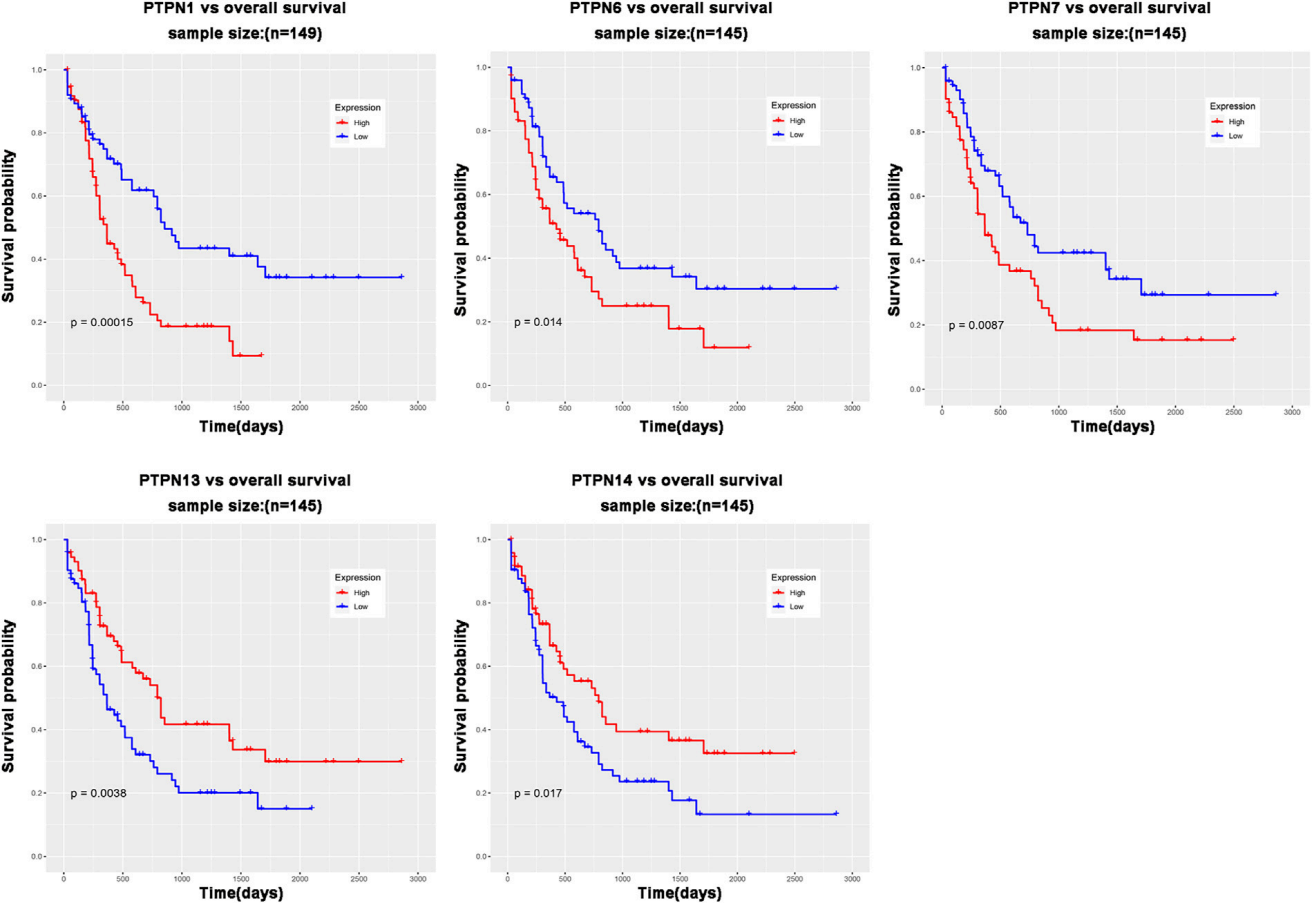
PTPN genes whose expression level is related to patient prognosis in AML patients.

The role of *PTPN* genes in AML patients’ survival was the subject of our further research. We conducted a prognostic analysis of *PTPN* genes in patients with AML using the LinkedOmics database. The results of the study showed that there was a significant correlation between poor overall survival (OS, *p* < .05, Figure 3) and increased expression levels of *PTPN1, PTPN6,* and *PTPN7* in all AML patients, but was associated with low expression of *PTPN13* and *PTPN14* (OS, *p* < .05, Figure 3). However, there were no significantly different in the expression levels of *PTPN2, PTPN3, PTPN4, PTPN5, PTPN9, PTPN11, PTPN12, PTPN18, PTPN20, PTPN21, PTPN22*, and *PTPN23* (Supplementary Figure S3). *PTPN1, PTPN6*, and *PTPN7* overexpression may indicate a poor prognosis for AML.

### Clinical samples were used to verify the mRNA and protein levels of *PTPNs* in AML and normal samples

**FIGURE 4:**
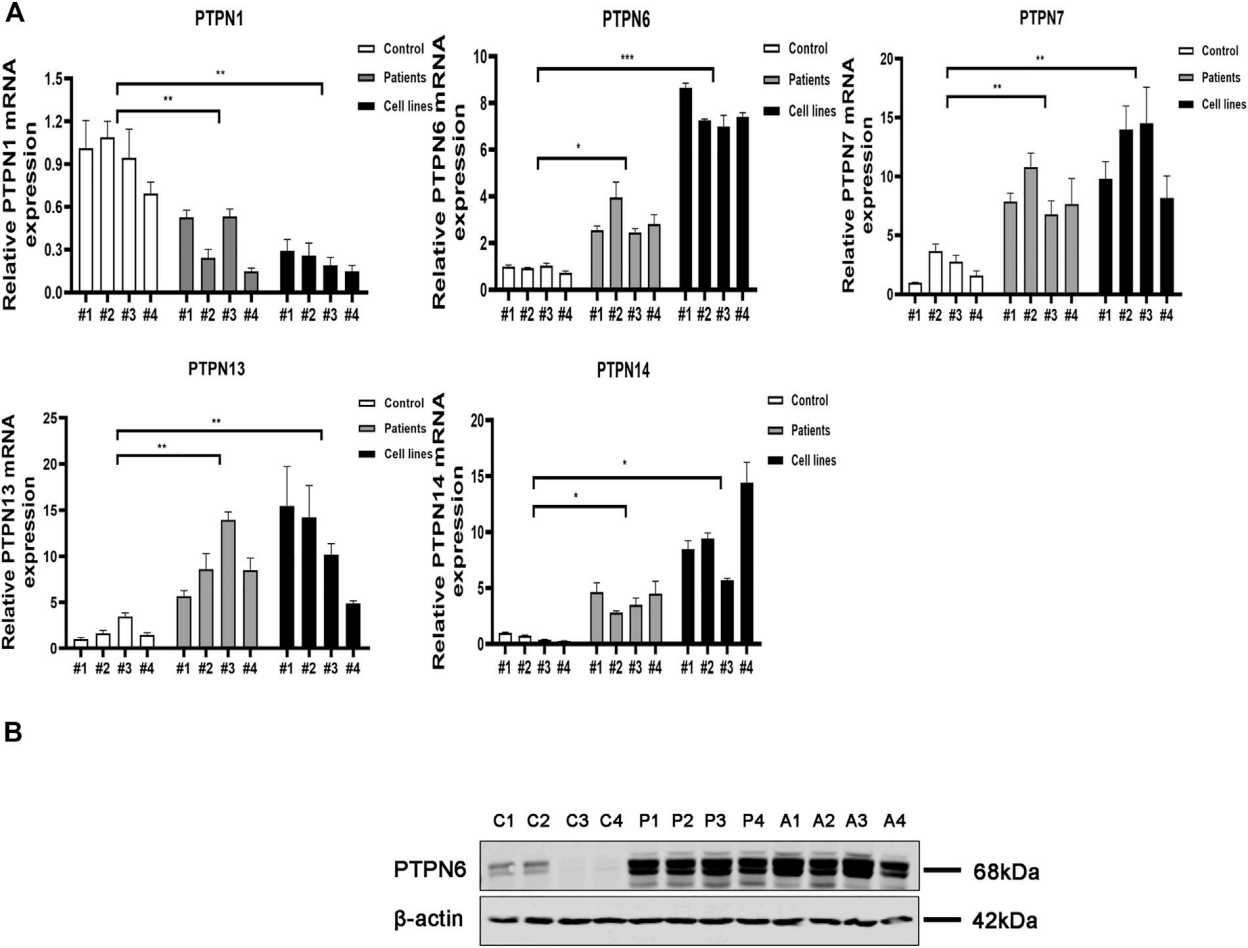
Relative mRNA and protein expression of the PTPN gene in AML samples and AML cell lines and normal controls were detected by qRT-PCR and western blotting. **(A)** Relative mRNA expression of PTPN gene in AML samples and AML cell lines and normal healthy controls detected by qRT-PCR. **(B)** Protein expression levels of PTPN6 in AML samples and AML cell lines and normal healthy controls detected by western blotting. Note: C1-C4 represent 4 normal controls, P1-P4 control 4 AML clinical samples, A1-A4 represent 4 AML cell lines including HL-60, KG-1, THP-1, and MOLM-13.

We next carried out qRT-PCR experiments utilizing clinical samples and cell lines to confirm further the expression level of *PTPNs* mRNA in AML. According to the findings, the expression of *PTPN 6, PTPN 7, PTPN 13*, and *PTPN 14* was higher in AML patients (*p* = .0116, *p* = .0034, *p* = .0092, and *p* = .0057, respectively) and AML cell lines (*p* = .0004, *p* = .0035, *p* = .0357, and *p* = .0177, respectively) than in normal individuals. The expression level of *PTPN1* was, however, lower in AML patients (*p* = .0094) and AML cell lines (*p* = .0013) (Figure 4A). However, there were no significantly different in the expression levels of *PTPN2, PTPN3, PTPN4, PTPN5, PTPN9, PTPN11, PTPN12, PTPN18, PTPN20, PTPN21, PTPN22*, and *PTPN23* (Supplementary Figure S4A).

According to the results of GEO, GEPIA, LinkedOmics, and qRT-PCR, only *PTPN6* expression was increased in AML patients in all major databases and qRT-PCR, and the results were consistent with each other. Next, we explored the protein expression of PTPN 1, PTPN6, PTPN 7, PTPN 13, and PTPN 14 in normal controls, clinical AML patients, and AML cell lines. The results of western blotting assays showed that the expression of PTPN6 in AML patient samples and AML cell lines was significantly higher than that in normal controls (Figure 4B). However, the expression levels of PTPN1, PTPN7, PTPN13 and PTPN14 were not different among AML patient samples, AML cell lines and normal controls (Supplementary Figure S4B).

### Associations between genome-wide expression profiles and *PTPN6* expression

**FIGURE 5:**
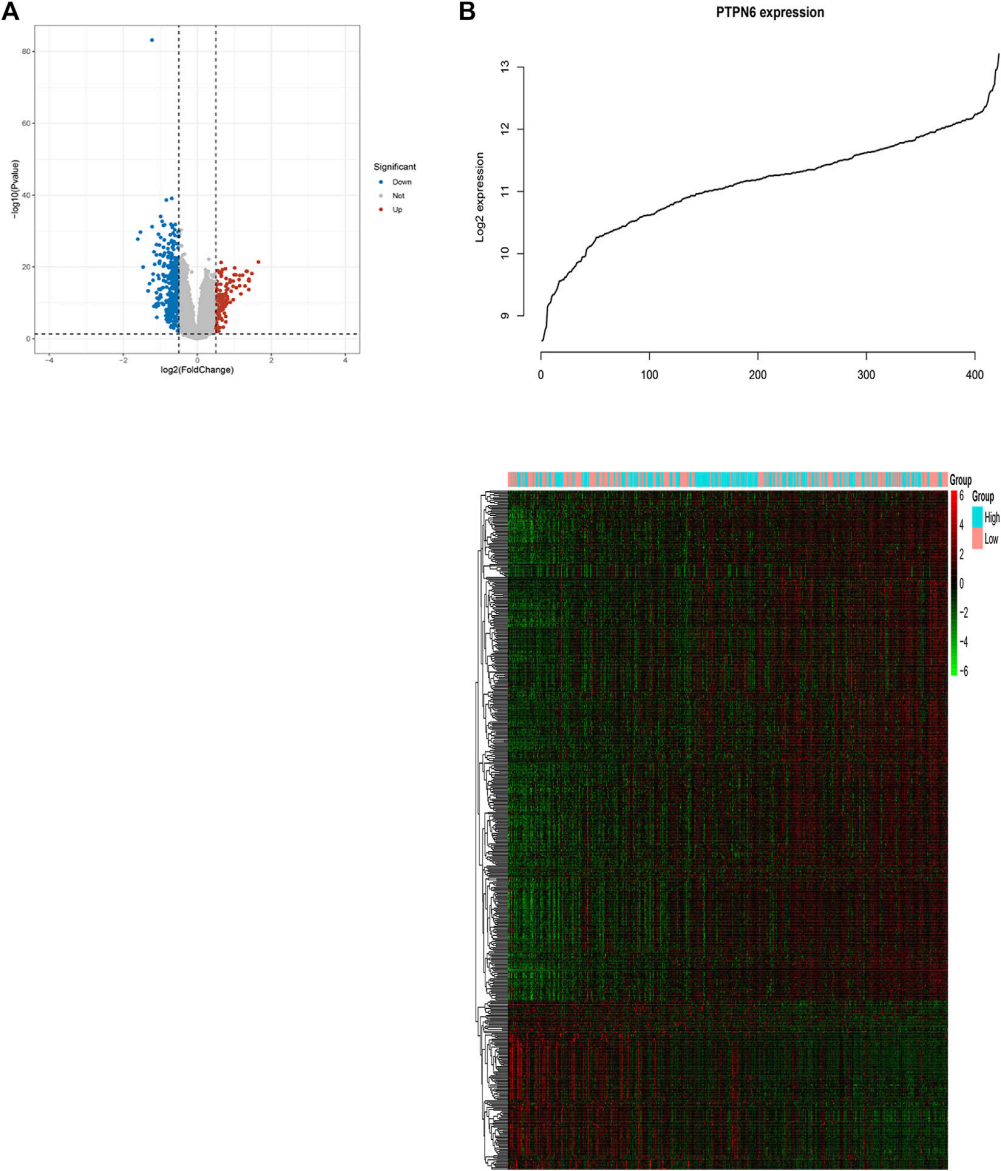
Genome-wide genes associated with PTPN6 expression. **(A)** Volcano plot of differential gene profiles between PTPN6 high and PTPN6 low. **(B)** Expression heatmap of PTPN6-associated genes. The top curve shows PTPN6’s expression distribution of 156 CN-AML samples.

In order to further study the biological role of *PTPN6* in leukemogenesis, the gene expression profile related to *PTPN6* was obtained based on the analysis of GSE dataset 37,642. As a result, 128 upregulated genes and 390 downregulated genes were identified as being significantly associated with the expression of *PTPN6* (fdr-adjusted *p* < .05 and FC > 1.5 or FC < 1/1.5, Figure 5A). In addition, we also presented these differentially expressed genes as a heat map (Figure 5B). The upregulated genes include: 1) Genes related to leukemia (such as *HHEX, NET1*), tumor-promoting factors (such as *CDK6, HOX* family genes), tyrosine kinase genes (*c-KIT, GRB10*); 2) Prognosis-related genes (such as *WT1, CXXC5, MSI2*, etc.); 3) *CD34* (a marker of hematopoietic progenitor cells); 4) AML drug resistance-related genes (such as *IGFBP2 and ABCC1*). Downregulated genes include: 1) Immune system activators, such as *CD86*; 2) blood tumor suppressors *ID2* and *KLF4*; 3) *CEBPB*, *CEBPB* is a *BCR/ABL* negative regulator gene, which can inhibit the proliferation of BCR/ABL-positive cells, and promote cell differentiation.

### Genetic alterations and correlations of *PTPN* genes in AML

**FIGURE 6:**
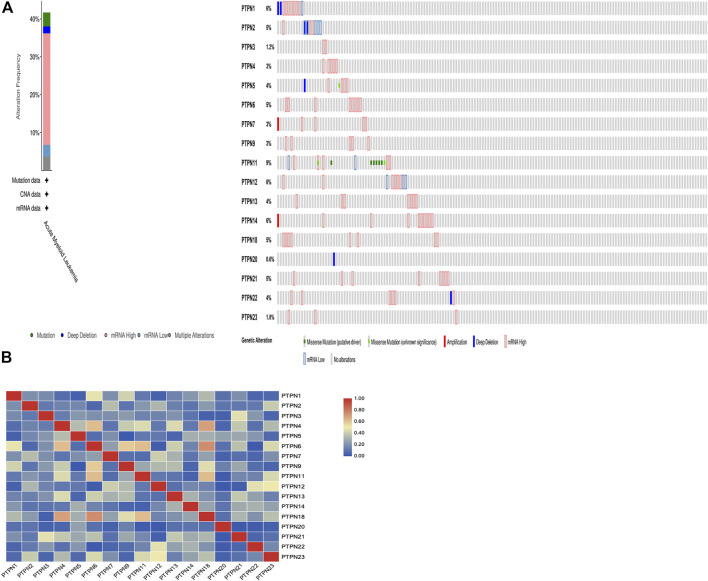
Genetic alteration and correlations of PTPN genes in AML. **(A)** Genetic alteration of PTPN gene in AML. **(B)** Genetic correlations of PTPN genes in AML.

We gathered details on genetic changes to the PTPN genes and determined any gene-to-gene correlations using the online resource cBioPortal and the “TCGA, Firehose Legacy” database for AML. In 68/163 (42%) patient samples with AML, *PTPN* genes were changed (Figure 6A). Mutation, deep deletion, mRNA overexpression, mRNA down-expression, and numerous alterations were among the several genetic alterations. The percentage of genetic alterations in *PTPN* family members for leukemia varied from .6% to 9% for individual genes based on the TCGA, Firehose Legacy dataset (*PTPN1*, 6%; *PTPN2*, 5%; *PTPN3*, 1.2%; *PTPN4*, 3%; *PTPN5*, 4%; *PTPN6*, 5%; *PTPN7*, 3%; *PTPN9*, 3%; *PTPN11*, 9%; *PTPN12*, 6%; *PTPN13*, 4%; *PTPN14*, 6%; *PTPN18*, 5%; *PTPN20*, .6%; *PTPN21*, 5%; *PTPN22*, 4%; *PTPN23*, 1.8%; Figure 6A). In addition, cBioPortal was used to investigate the expression of *PTPN* genes in AML [using mRNA sequencing (RNA-seq) version V2 RSEM], and the relationships between certain *PTPN* genes (including Pearson’s correlation) were calculated. Except for *PTPN1* and *PTPN22*, the findings showed a significant positive correlation between any two *PTPN* family gene members (Pearson = .00952, *p* = .905) (Figure 6B).

### Function and interaction of *PTPN* family genes

**FIGURE 7:**
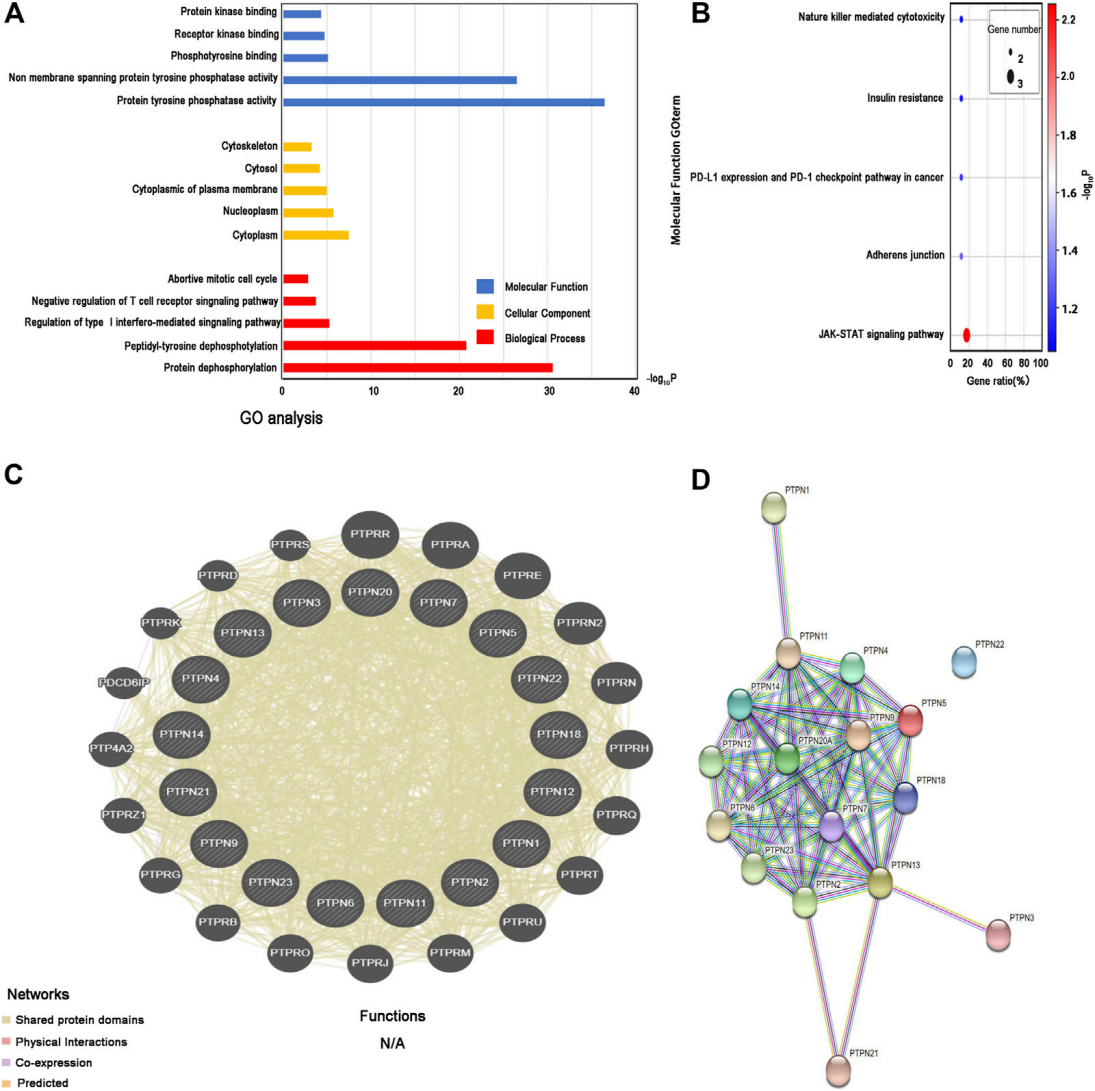
Enrichment and correlation analysis among PTPN family genes. **(A)** GO analysis of PTPN family genes. The top five enriched categories for Molecular Function, Cellular Component, and Biological Process were showed. **(B)** KEGG pathway analysis of PTPN family genes, top five KEGG pathway category were showed. **(C)** Gene–gene interaction network among PTPN gene family members. **(D)** Protein–protein interaction network among PTPN gene family members.

The three main categories of GO analysis were molecular function groups, cellular component groups, and biological process groups. Figure 7A displays the top five enriched categories for each group as determined by the analysis results. GO analysis revealed that most PTPN proteins were associated with the cytoplasm. Protein dephosphorylation and protein tyrosine phosphatase activity were the main targets of *PTPN* genes’ actions. The KEGG pathway enriched 11 genes using the David online platform. JAK-STAT signaling pathway, Adherens junction, PD-L1 expression, and PD-1 checkpoint pathway in cancer, as well as insulin resistance and natural killer cell-mediated cytotoxicity, were the principal pathways associated with the eleven genes (Figure 7B). To further understand the relationships between colocalization, shared protein domains, co-expression, prediction, and pathways, interaction analysis of *PTPN* genes at the gene level was carried out using GeneMANIA (Figure 7C). The STRING protein-protein interaction network analysis revealed that the connections between the members of the *PTPN* gene family were complex (Figure 7D).

## Discussion

According to the Global Burden of Disease study, acute myeloid leukemia (AML) is the deadliest form of leukemia in the world, with 147,000 fatalities attributed to it in 2019 (Fitzmaurice et al., 2019; GBD 2019 Diseases and Injuries Collaborators, 2020) AML is linked to 40% of leukemia-related mortality in the United States and around the world, but only about 30% of leukemia occurrences (Siegel et al., 2021). AML incidence has increased more during the previous 10 years than chronic lymphocytic leukemia (CLL) (34% vs 25%), acute lymphoblastic leukemia (ALL) (22%), and chronic myeloid leukemia (13%), in part due to population expansion and aging as well as an increase in the standardized incidence rate (Cao et al., 2021). Therefore, it is noteworthy and crucial for AML that new biological therapeutic targets are found.

Together with protein-tyrosine kinases, PTPN regulates tyrosine phosphorylation and dephosphorylation in cellular signal transmission. They are essential members of the protein tyrosine phosphatases family (Tonks, 2006). Numerous studies have examined the relationships between specific *PTPN* family members and various neoplasms (Chen et al., 2020; Mitra and Ayyannan, 2020). To date, no report has provided an overview of the connections between *PTPN* family genes and human leukemia. In this study, *PTPN* gene expression patterns were illustrated for the first time, and it was also shown how the *PTPN* family of genes and AML diagnosis and prognosis are related. This information gave genes clearer understanding of the clinical value of all the *PTPN* genes in human leukemia.

We investigated the level of PTPN gene expression in AML using GEO database and the online databases GEPIA, Next, we used the LinkedOmics database to explore the relationship between the *PTPN* family’s expression and AML patients’ survival. Then we validated our findings using qRT-PCR. As a result of the high levels of expression of numerous *PTPN* family members in human AML discovered in our study, *PTPNs* may 1 day be used as an AML diagnostic biomarker. However, there is much debate about the expression of the *PTPN* family in AML and normal samples. The expression level of *PTPN1* in AML mice is significantly lower than in normal mice, and myeloid-specific deficiency of *PTPN1* can promote the development of AML (Le Sommer et al., 2018). In the GSE149237 microarray, there was no difference in the expression of *PTPN1* in AML and normal samples. In contrast to the GEPIA database and RT-PCR, which revealed the opposite findings. This suggests that the expression and role of *PTPN1* in AML are still controversial, and further studies are needed to explore and clarify. *PTPN2* is frequently absent in adult and pediatric T-ALL patients, but the expression level of *PTPN1* does not correlate with patient prognosis (Alcantara et al., 2019). Similar to our findings, *PTPN2* expression did not differ between AML and normal samples and was not associated with patient prognosis. *PTPN3* is an oncoprotein with a poor prognosis and can promote cell invasion and proliferation in biliary cancer (Wang et al., 2018). *PTPN4* is highly expressed in AML and can guide the classification of AML (Kabir et al., 2013). *PTPN5* act as a tumor suppressor in breast cancer (Palaniappan et al., 2018), but previous studies have not found differences in the expression of *PTPN5* between AML and normal samples. Our findings demonstrated that, *PTPN3, PTPN4*, and *PTPN5* were not differentially expressed in AML and normal blood, and there was no correlation between their expression levels and patient survival. Several transmembrane receptors’ intracellular signaling is modulated by *PTPN6* (SHP-1). When SHP-1 expression and activity are reduced, JAK kinase activity is raised, which causes aberrant cell proliferation (Wu et al., 2003). Activation of *PTPN6* (SHP-1) recruits CAMK1 to mediate self-renewal of AML (Hao et al., 2021). Compared to samples from AML patients and AML cell lines, the expression of *PTPN6* was significantly lower in our study’s normal samples (whether in GEO and GEPIA databases or RT-PCR and western blotting results). Further evidence that *PTPN6* can be employed as a marker for the diagnosis and prognosis of AML comes from the substantial correlation between the high expression of *PTPN6* and the poor survival of AML patients. Cytoplasmic protein tyrosine phosphatase PTPN7, sometimes referred to as hematopoietic PTP, was first cloned from human T-cells. Since PTPN7 dephosphorylated ERK, T-cell activation was decreased in T cells derived from *PTPN7*-KO mice, as seen by the hyperphosphorylation of ERK on those cells (Inamdar et al., 2019). Our results from GEO and RT-PCR showed that *PTPN7* expression was lower in normal samples than in AML patients. However, the GEPIA results showed no statistically significant difference between the two. In contrast, the cytoplasmic PTP PTPN9 is broadly distributed in the tissues of the brain, leukocytes, endocrine cells, and other cells (Wang et al., 2019). PTPN11 is involved in many signal transduction functions necessary for normal hematopoiesis, and mutations in *PTPN11* can mediate the development of AML and are associated with poor prognosis (Alfayez et al., 2021). *PTPN12* is highly expressed in AML samples (Arora et al., 2012). Our results showed that in the GSE149237 microarray dataset, the expression of *PTPN11* was higher in AML than in normal controls, but in the GEPIA database, there was no difference between the two. The expression of *PTPN9* and *PTPN12* did not differ between AML patients and normal controls, neither in the GEO nor the GEPIA database. Transcription levels of *PTPN13* are abnormally elevated in myeloid malignancies (Mundle et al., 1999). *PTPN14* is now considered a tumor suppressor, but its expression and role in AML have not been studied and reported (Au et al., 2010). Our results showed the opposite. In the analysis of the GEO database, *PTPN13* was higher in AML than normal controls, while *PTPN14* was the utter opposite. In contrast, GEPIA results showed *PTPN13* and *PTPN14* expression in AML and normal controls with no difference. RT-PCR results showed that the expression of *PTPN13* and *PTPN14* was higher in AML and AML cell lines than in the control group. Only brain tissues express *PTPN20* (Xia et al., 2018), and overexpression of *PTPN21* promotes the proliferation of ALL cells by activating the mitogen-activated protein kinase (MAPK) signaling pathway (Wang et al., 2020). In our study, *PTPN20* expression did not differ between AML and normal samples. However, the expression results of *PTPN21* in AML and normal samples in the GSE149237 microarray dataset and GEPIA database were inconsistent. *PTPN23* is required for AML cell survival (Zhang et al., 2017). However, our results showed that normal samples had higher expression levels of *PTPN23* than AML samples. Furthermore, the expression of *PTPN23* in AML patients was not associated with the prognosis of the patients. Combined with the GSE149237 microarray dataset, GEPIA database, LinkedOmics database, RT-PCR, and western blotting results. *PTPN6* can be used as a marker for the diagnosis and prognosis of AML. However, further research and testing are needed to determine its association with AML risk.

Significant genomic genes of genetic alterations include changing the genetic code, inducing gene disruptions, and producing phenotypic differences (Egger et al., 2004; Zhou et al., 2016; Wu and Xu, 2020). An abnormal expression and *PTPN* dysfunction in AML can result from altered *PTPN* genes’ chromosomal structure. In our study, 68/163 (42%) of the patient samples with AML had *PTPN* gene alterations, which included mutations, deep deletions, mRNA overexpression, mRNA down-expression, and multiple alterations. *PTPN11* mutations are frequently associated with acute myelomonocytic/monocytic leukemia subtypes, and *PTPN11* is associated with lower rates of complete remission and shorter overall survival (Alfayez et al., 2021). Our study shows that *PTPN11* has the highest probability of mutation, followed by *PTPN1, PTPN12, PTPN12*, and *PTPN6*. These results suggest that high genetic alterations in the *PTPN* gene are related to the development of AML.

GO analysis in this study showed that the PTPN protein was primarily related with the cytoplasm. The primary functions of *PTPN* genes, which have been extensively documented in various publications, are protein tyrosine phosphatase activity and dephosphorylation. *PTPN11* contains two N-terminal Src homology 2 (SH2) domains, a protein tyrosine phosphatase (PTP) catalytic domain, and a COOH terminus. *PTPN11* further contributes to the transformation of AML by encoding a ubiquitously expressed cytoplasmic phosphatase SHP2, which mediates cellular responses to hormones and cytokines (Stasik et al., 2021). Additionally, the outcomes of interaction network analysis at the gene and protein levels further suggested extensive interactions between *PTPN* members and other genes. However, few studies have examined interactions between *PTPN* members in AML. According to one investigation, the dephosphorylation of IRF3 at Y245 is mediated by a protein that both *PTPN1* and *PTPN2* target (Xia et al., 2019). By dephosphorylating protein tyrosine kinases unique to lymphocytes, *PTPN2* and *PTPN22* have been found to affect T-cell receptor signaling (Cloutier and Veillette, 1999; Wiede et al., 2011). Multiple studies showed that PTPN5 and PTPN7 might bind to and inactivate the mitogen-activated protein kinases Erk2 and P38, which could negatively influence cell proliferation and differentiation (Francis et al., 2011; Francis et al., 2013; Francis et al., 2014). It has been noted that diffuse large B-cell lymphomas are prevented from progressing by the hypermethylation of the *PTPN6* and *PTPN13* promoters (Wang et al., 2016). In our study, the prognosis of patients with AML was related to *PTPN6, PTPN7, PTPN13*, and *PTPN13*. Therefore, *PTPN* members have the potential to play the role of numerous diseases, including AML, *via* consortium processes.

Our results demonstrate the expression status and prognostic value of *PTPN* members in AML. The results showed the differential expression of some *PTPN* members and their correlation with the prognosis of AML. These members’ samples from healthy people had significantly lower levels of *PTPN6* expression than samples from AML patients. Furthermore, patients with AML had significantly worse survival rates when their PTPN6 expression was higher. *PTPN6* may be employed as a diagnostic and prognostic marker for AML, according to our findings. To further develop the therapeutic applicability of PTPNs, further well-designed studies are required to explain the importance of our findings.

## Data Availability

The datasets presented in this study can be found in online repositories. The names of the repository/repositories and accession number(s) can be found in the article/Supplementary Material.
