# Supplementary material for: Comprehensive analysis of PTPN family expression and prognosis in acute myeloid leukemia
Source: Front Genet. 2023 Jan 9;13:1087938. doi: 10.3389/fgene.2022.1087938 (PMC9868563; doi:10.3389/fgene.2022.1087938)
Supplement: Supplementary file 1 [file Table1.DOCX]

**Supplement table. The sequences of primers used for qRT-PCR.**

| **Primer names** | **Primer sequences** |
| --- | --- |
| PTPN1 | Forward: AGTTGCCTTGTTGAAGGGGAT |
|  | Reverse: AGAAGGTGTCAAGATGGGTGG |
| PTPN2 | Forward: AGCGGGAGTTCGAAGAGTTG |
|  | Reverse: TGTTAGGAAGTGGACCCTGTG |
| PTPN3 | Forward: AGCGACAGAAACAGGCTGAA |
|  | Reverse: TTCCGAGAGCCCATAGTCCA |
| PTPN4 | Forward: ATTCCCTGCGGGAGTCAATG |
|  | Reverse: ACCCGTGTGGCATCATAAGG |
| PTPN5 | Forward: TGAGAAAGCTTCCTGCCCTG |
|  | Reverse: TCTCTCTCACTCCTGGCTCC |
| PTPN6 | Forward: GGAGAAGTTTGCGACTCTGAC |
|  | Reverse: GCGGGTACTTGAGGTGGATG |
| PTPN7 | Forward: CTACATCCGAGGCTATGACGG |
|  | Reverse: GGCCAGTAGTGGACACATTTC |
| PTPN9 | Forward: TTGTCATGACCACCCGCTTT |
|  | Reverse: AGTCTGGCCAGCTCAAGAAC |
| PTPN11 | Forward: GCCTGCAAAACACGGTGAAT |
|  | Reverse: TCCCCTGGAGTAGAGCTTGT |
| PTPN12 | Forward: CAGATCGTGGCTGACAGAGG |
|  | Reverse: TGGCCCATAGACGCCCTTTA |
| PTPN13 | Forward: TTGGAATGACACTGTATTGGGG |
|  | Reverse: CCAAGCAGTATGCTGTTGAGAT |
| PTPN14 | Forward: AGCTGCGAGAGACGCACTA |
|  | Reverse: GCTCATTAGCGAATTTGTCCAGA |
| PTPN18 | Forward: GGCGAGTTCAGCAAAAGGTG |
|  | Reverse: GTGGACACAGAGGGGTTCAG |
| PTPN20 | Forward: CTAGGCGTGGACCCAACT |
|  | Reverse: CAATTTTTGTTCACCTGGGCCT |
| PTPN21 | Forward: TGACAGCAGAAGAGGAGGGT |
|  | Reverse: TGTTGTGCCTGGAACCAAGT |
| PTPN22 | Forward: GCCACCCAGGGTCCTTTATC |
|  | Reverse: AGCGCTCACACTTTTTCTTTCC |
| PTPN23 | Forward: TGGAGTTGCTCAGACAGGACA |
|  | Reverse: CGCACTGGAAATGGGTACAG |
| GAPDH | Forward: GCAAATTCCATGGCACCGTC |
|  | Reverse: TCGCCCCACTTGATTTTGG |
