# Supplementary figures and images for: Comprehensive analysis of PTPN family expression and prognosis in acute myeloid leukemia
Source: Front Genet. 2023 Jan 9;13:1087938. doi: 10.3389/fgene.2022.1087938 (PMC9868563; doi:10.3389/fgene.2022.1087938)

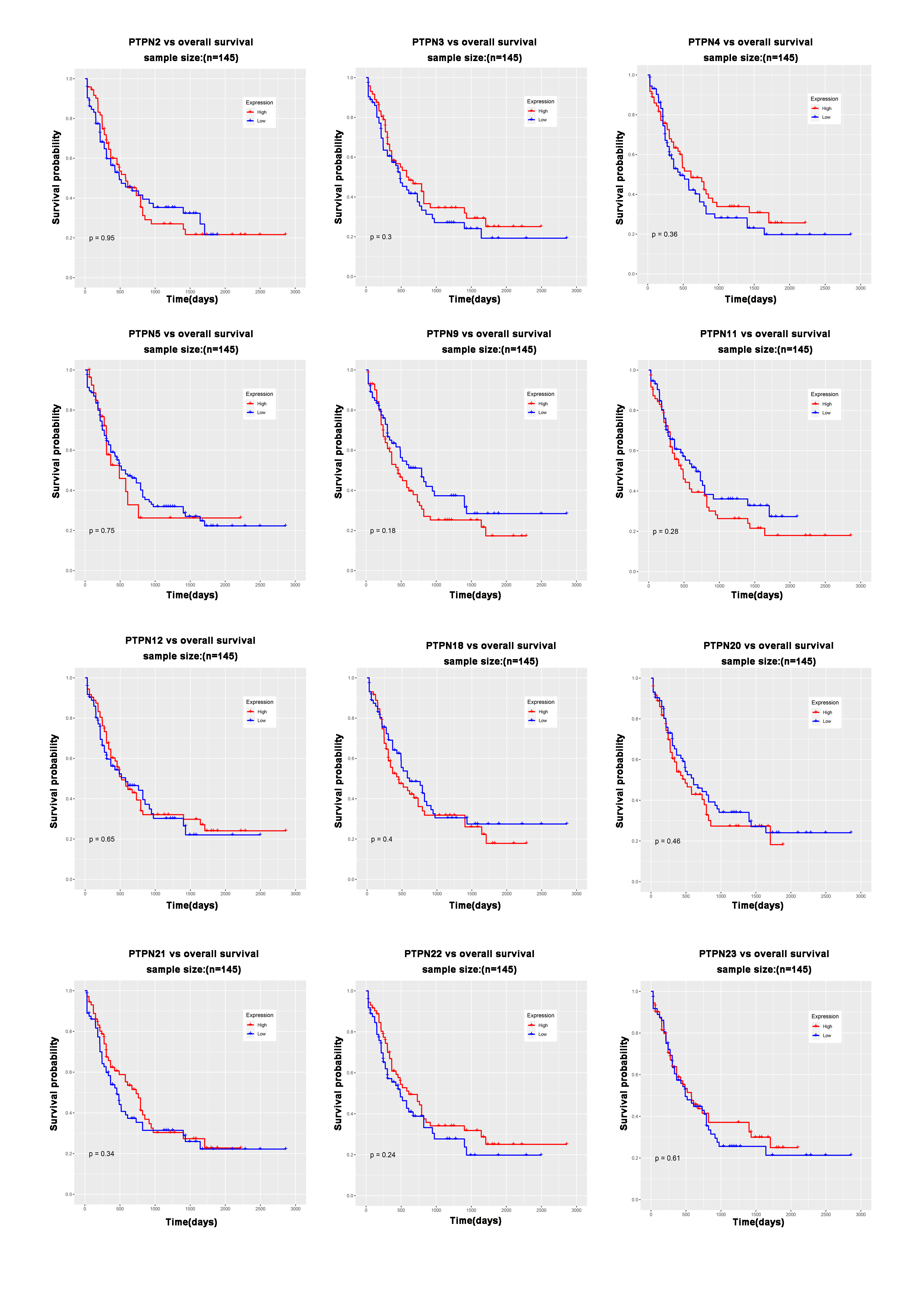

Supplement: Supplementary file 2 [file Image3.TIF]

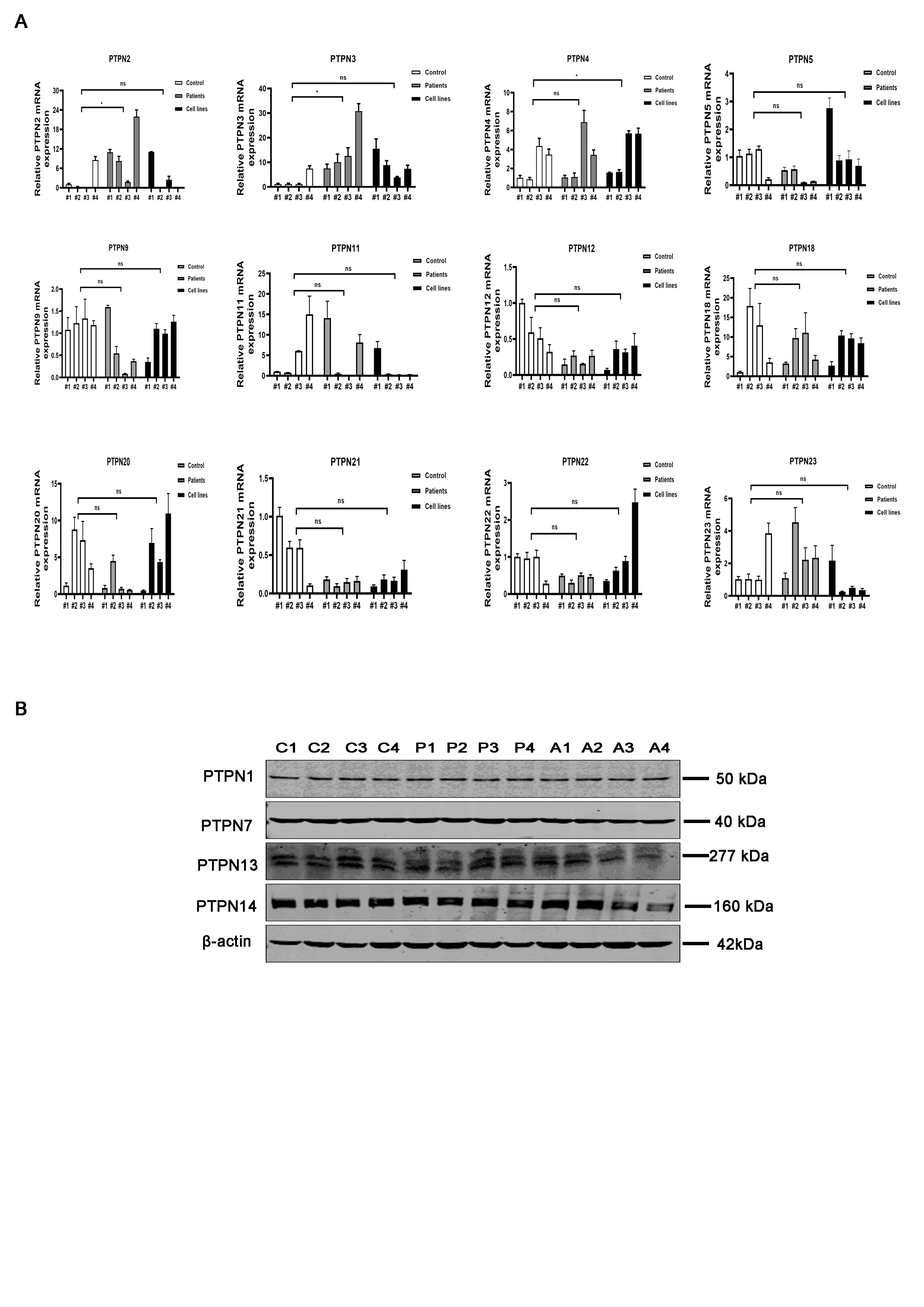

Supplement: Supplementary file 3 [file Image4.TIF]

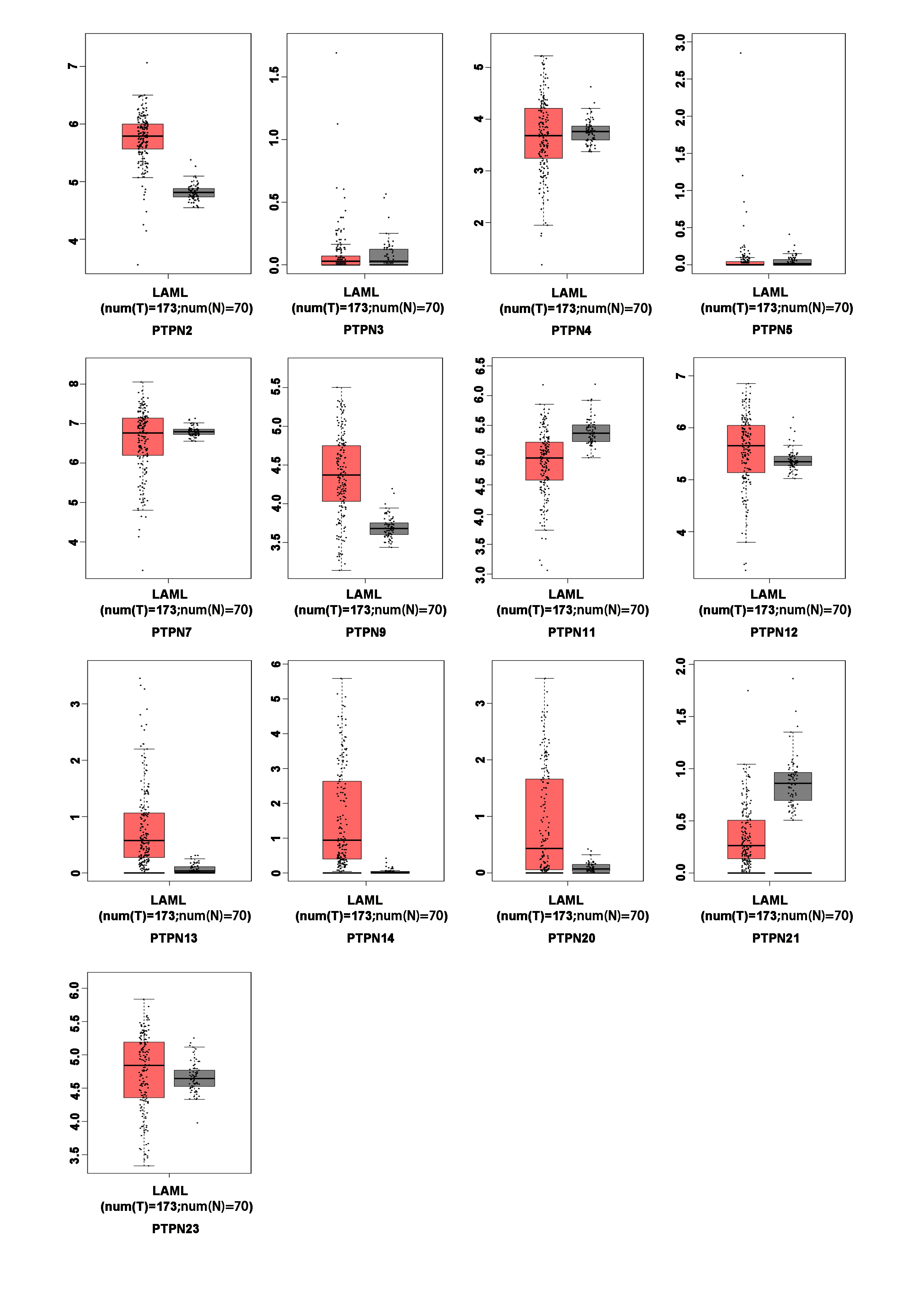

Supplement: Supplementary file 4 [file Image2.TIF]

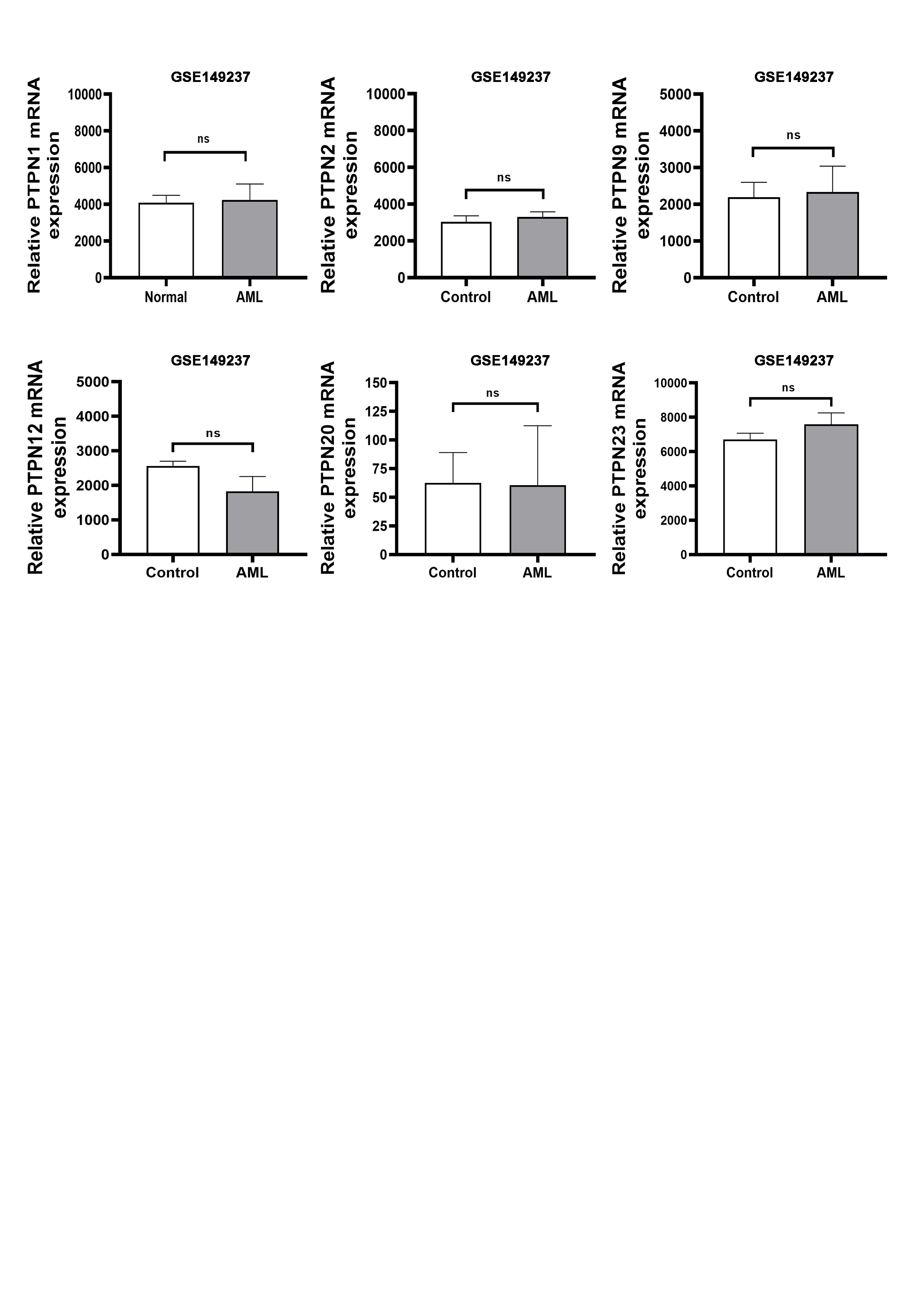

Supplement: Supplementary file 5 [file Image1.TIF]
